# Moderate exercise improves function and increases adiponectin in the *mdx* mouse model of muscular dystrophy

**DOI:** 10.1038/s41598-019-42203-z

**Published:** 2019-04-08

**Authors:** Aaron S. Zelikovich, Mattia Quattrocelli, Isabella M. Salamone, Nancy L. Kuntz, Elizabeth M. McNally

**Affiliations:** 1 Center for Genetic Medicine, Feinberg School of Medicine, Northwestern University, Chicago, IL USA; 2 Ann and Robert H. Lurie Children’s Hospital of Chicago, Chicago, IL USA; 3 Indiana University School of Medicine, Indianapolis, IN USA

## Abstract

The loss of dystrophin produces a mechanically fragile sarcolemma, causing muscle membrane disruption and muscle loss. The degree to which exercise alters muscular dystrophy has been evaluated in humans with Duchenne Muscular Dystrophy (DMD) and in mouse models including the *mdx* mouse but with inconsistent findings. We now examined two different levels of exercise, moderate and low intensity, in the *mdx* mouse model in the DBA2J background. *mdx* mice at 4–5 months of age were subjected to two different doses of exercise. We found a dose-dependent benefit for low and moderate exercise, defined as 4 m/min or 8 m/min, for 30 minutes three times a week. After six months, exercised *mdx* mice showed improved tetanic and specific force compared to the sedentary group. We also observed increased respiratory capacity manifesting as greater minute volume, as well as enhanced cardiac function mitigating the decline of fractional shortening that is normally seen. Exercised *mdx* mice also showed a dose-dependent increase in serum adiponectin with a concomitant reduced adipocyte cross sectional area. These findings identify moderate intensity exercise as a means to improve muscle performance in the *mdx* DBA2J mice and suggest serum adiponectin as a biomarker for beneficial exercise effect in DMD.

## Introduction

Duchenne Muscular Dystrophy (DMD) is an X-linked genetic disorder that causes loss of muscle function beginning in early childhood. DMD arises from genetic mutations that result in loss of dystrophin protein^1^, and this lack of dystrophin disrupts the dystrophin-glycoprotein complex causing instability of the sarcolemma in cardiac and skeletal muscle cells. Boys with DMD experience a sharp decline in ambulatory capacity between the ages of 7–10 years with the majority using a wheelchair full time by the early teenage years. Aerobic exercise has been hypothesized to improve muscle strength and prevent contractures in boys with DMD^2^. However, the overall impact and beneficial mechanisms enabled by aerobic exercise regimens on dystrophic muscle even in animal models requires further study.

Exercise studies in the *mdx* mouse model have yielded inconsistent results. High intensity exercise has been suggested to exacerbate dystrophic pathology, but in the context of dystrophy, low intensity exercise may actually be beneficial^3–9^. Historically, *mdx* mice have been subjected to exercise at high speeds, typically between 12–23 meters/minute (m/min), on a downhill treadmill^9^. Under these conditions, which physiologically mimic lengthening contractions, *mdx* muscle has often shown functional impairment, as indicated by decline in grip strength^10^, histopathological signs of worsening injury^11^, and even a decrease of genes usually expressed in response to exercise, like *Pgc1a*^12^. The adverse response to exercise in the *mdx* mouse is described in the TREAT-NMD standardized protocol as 12 m/min for 30 minutes twice a week, and this speed, duration and frequency are intended to worsen the phenotype (Protocol: Treat-NMD DMD_M.2.1.001).

The *mdx* mouse has been most commonly studied in the C57Bl10 background. However, the identical dystrophin exon 23 premature stop codon in the DBA2J background has been shown to intensify the dystrophic phenotype resulting in functional deficits closer at an earlier age than in the C57Bl10 background^13–16^. We used this model of *mdx* mice in the DBA2J background to evaluate two different levels of aerobic exercise. Dystrophic mice were exercised with 8 m/min (moderate intensity) and 4 m/min (low intensity) treadmill exercise regimens, and notably both these regimens are less than the 12 m/min (high intensity) regimen that associates with a worsening of the *mdx* phenotype. We assessed exercise-induced changes in not only locomotory skeletal muscles, but also respiratory muscles, cardiac muscle and adipose tissue. With moderate and low intensity exercise, *mdx* mice exhibited an improvement of skeletal muscle function, attenuation of cardiac dysfunction, and improvement in respiratory capacity, and generally did so in a dose-dependent manner. Thus, moderate exercise was beneficial in dystrophic mice without increasing muscle membrane damage or fibrosis. Moderate exercise reduced adipocyte cross sectional area and increased serum adiponectin levels, suggesting adiponectin as a potentially useful biomarker for exercise in DMD.

## Results

### Exercise improved skeletal muscle function in mdx mice

**Figure 1:**
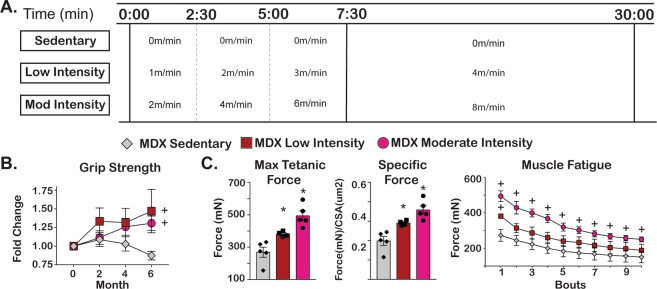
Exercise improved skeletal muscle function in *mdx* mice. Four month-old male *mdx* mice from the DBA2J background (n = 5/cohort) were exercised three times per week for 24 weeks between 8 am–12 pm. (**A**) The exercise protocol included a 7 min 30 second warm-up with an increase in speed every 2 minutes and 30 seconds until maximum speed was attained. Mice remained on treadmill for additional 22 minutes and 30 seconds at maximum speed for a total of 30 minutes duration. Three levels of exercise were evaluated: 0 m/min (sedentary), 4 m/min (low intensity) and 8 m/min (moderate intensity). (**B**) Average forelimb grip strength increased in both exercise groups whereas it declined in sedentary group. (**C**) Maximum tetanic force and specific force was determined for the *Tibialis anterior* (TA) muscles and showed an intensity-dependent improvement in muscle function that was sustained over 10 consecutive bouts of isometric contraction. Histograms depict single values and mean ± s.e.m.; curves depict mean ± s.e.m.; box plots depict 10–90% distribution with mean(+); *P < 0.05 vs sedentary; 1-way ANOVA test with Tukey’s multiple comparison; + , P < 0.05 vs vehicle, 2-way ANOVA test with Tukey’s multiple comparison.

To examine how varying thresholds of aerobic exercise affect dystrophic muscle, we subjected *mdx* mice to treadmill running over a six-month interval, evaluating two different running speeds. These studies were conducted on mice with established disease with exercise beginning at 4–5 months of age. To avoid the deleterious effects of downhill running on muscle integrity, mice ran only on a horizontal platform. Male *mdx* mice from the DBA/2 J background were evaluated, as this background confers more severe functional deficits than other strains, such as 129T2 and C57Bl/6^13,16^. At study onset *mdx* mice were assigned to the sedentary (0 m/min), low intensity (4 m/min), or moderate intensity (8 m/min) groups (n = 5 per group). Each exercise session consisted of a 7 minute 30 second warm up period followed by a 22 minute 30 second interval at target speed (Fig. 1A). This exercise program was repeated three times per week for six months. One mouse in the low intensity cohort died at the end of month four, and the remaining mice in all cohorts completed the six month protocol.

To assess the effect of exercise on muscle function in dystrophic mice, we analyzed grip strength during the study and *ex vivo* muscle mechanics at study endpoint. Grip strength was monitored every 8 weeks over study duration and at least 48 hours after most recent exercise interval. Bilateral grip strength was normalized to initial body weight (g). Both low and moderate intensity exercise cohorts experienced an increase in grip strength as compared to baseline. At the study endpoint, the fold change in low intensity cohort was 1.47 and the fold change in the moderate intensity cohort was 1.31 (Fig. 1B). Conversely, the sedentary group had a fold change of 0.87. At study endpoint, we evaluated isometric muscle mechanics of the *tibialis anterior (TA)* muscle. Chronic exercise improved maximum tetanic force in a dose-dependent fashion. The maximum force generated by sedentary TA muscle was 269 ± 31 mN, compared to low intensity exercised TA at 381 ± 8 mN and moderate intensity exercised TA at 494 ± 30 mN (Fig. 1C). Specific force, normalized to muscle cross sectional area, and resistance to muscle fatigue were also increased in both exercise groups (Fig. 1C). TA cross sectional area was similar between cohorts (sedentary 1357um^2^, low intensity 1311um^2^, and moderate intensity 1380 um^2^, Supplemental Fig. 1A). Fiber type analysis revealed a dose-dependent increase in the proportion of type 2a fibers as expected (Supplemental Fig. 1B). In addition, both exercise groups showed a modest increase in expression of the *Pgc1a* gene in the gastrocnemius muscle, a marker of aerobic exercise, as compared to the sedentary group (Supplemental Fig. 1C). Thus, the increase in oxidative myofiber fraction and *Pgc1a* transcript confirmed the expected remodeling of muscle to an increased oxidative profile in response to exercise-driven adaptation.

### Exercise improved respiratory function in mdx mice

**Figure 2:**
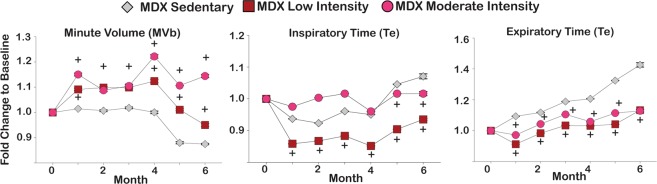
Exercise improved respiratory function in *mdx* mice. Whole-body plethysmography demonstrated an increase in respiratory minute volume in an intensity-dependent manner. Inspiratory (Ti) and expiratory (Te) times were inversely related to disease progression. Both exercise groups had an attenuation of increased Ti and Te as compared to sedentary group. Curves depict mean ± s.e.m.; + , P < 0.05 vs vehicle, 2-way ANOVA test with Tukey’s multiple comparison.

Respiratory function was assessed monthly in non-anesthetized mice using whole body plethysmography. Minute Volume (MVb), a surrogate endpoint for respiratory capacity, increased with aerobic exercise. At treatment end, the moderate intensity cohort had a 44% improvement in MVb as compared to baseline whereas the low intensity cohort had a 5% decline compared to baseline (Fig. 2). The sedentary group had a 13% decline, which reflects disease progression. Inspiratory time (Ti) and expiratory time (Te) are inversely related to disease progression. Ti increased by 7% in the sedentary group, decreased by 6% in the low intensity group, and increased by 1% in the moderate intensity group. Te increased by 43% in the sedentary group and increased by 13% in both the low and moderate intensity exercise cohorts (Fig. 2). Therefore, aerobic exercise improved respiratory capacity and attenuated the increase in Ti and Te of *mdx* mice.

### Exercise attenuated cardiac decline associated with disease progression in mdx mice

**Figure 3:**
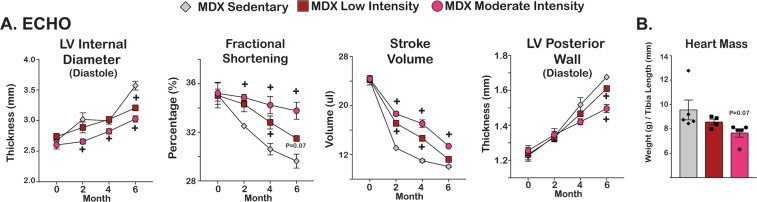
Exercise attenuated cardiac decline associated with disease progression in *mdx* mice. (**A**) Echocardiographic parameters indicated attenuation of cardiac decline associated with disease progression in exercised groups. Reduced left ventricular diameter, improved fractional shortening, and increased stroke volume were present after exercise. Hypertrophy associated with disease progression was attenuated with a smaller posterior wall thickness and (**B**) Heart mass showed a modest reduction in exercised mdx mice. Histograms depict single values and mean ± s.e.m.; curves depict mean ± s.d.; curves depict mean ± s.e.m.; *P < 0.05 vs sedentary; 1-way ANOVA test with Tukey’s multiple comparison; + , P < 0.05 vs sedentary, 2-way ANOVA test with Tukey’s multiple comparison.

Electrophysiological changes were assessed with monthly ambulatory electrocardiograms. Heart rates were consistently between 650–750 beats per minute throughout study duration and did not significantly differ between cohorts (Supplemental Fig. 2A). QRS duration remained consistent throughout study duration. QTc had a slight increase in the low intensity cohort at month 5, but was non-significant and did not continue at month 6. Echocardiography was performed every 2 months to assess cardiac function. Changes with cardiomyopathy and hypertrophy were assessed with Left Ventricle Internal Diameter during Diastole (LVIDD) and Left Ventricle Posterior Wall Thickness (LVPWT). At month 6, there was an exercise dose-dependent reduction in the thickness of the LVIDD and the LVPWT suggesting an attenuation of disease progression as compared to the sedentary cohort (Fig. 3A). Systolic function was tracked through fractional shortening and stroke volume. Fractional shortening and stroke volume had slower rates of decline in an exercise dependent manner in both the low intensity and moderate intensity cohorts. Heart mass was normalized to tibia length to account for differences in mouse size. There was a trend in the reduction of heart mass associated with increase exercise intensities (Fig. 3B). *Pgc1a* was increased in the hearts from both exercise groups as compared to the sedentary cohort (Supplemental Fig. 2B). Aerobic exercise delayed progression of cardiomyopathy without adversely affecting electrical function of the dystrophic heart.

### Exercise did not affect muscle and heart fibrosis in mdx mice

**Figure 4:**
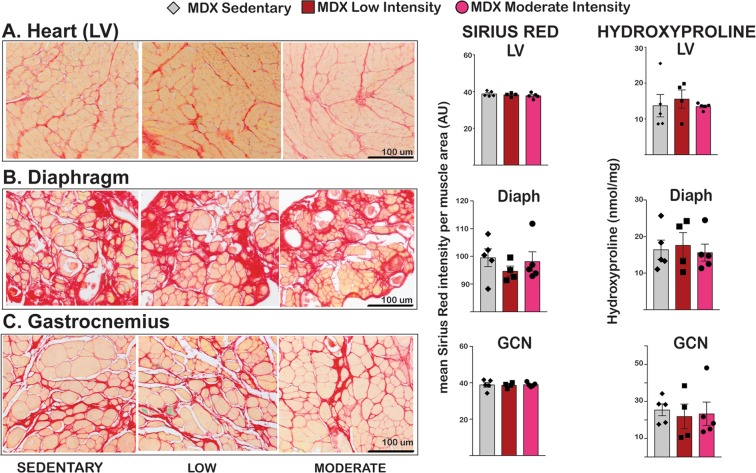
Exercise did not increase fibrosis in *mdx* mice. (**A–C**) Heart, diaphragm, and gastrocnemius showed no increase in Sirius red staining with exercise. Hydroxyproline content did not differ among groups confirming the histological findings.

We next evaluated the effect of these exercise regimens on fibrosis and muscle injury in *mdx* mice. Fibrosis was imaged and quantitated through picrosirius red staining and hydroxyproline measurements. Picrosirius red staining appeared qualitatively similar among the groups with similar number and size of fibrotic regions in heart, diaphragm and gastrocnemius muscles (Fig. 4, left). Quantitation of picrosirius red signal from histology images confirmed the similar trends (Fig. 4, center). Hydroxyproline levels were measured from tissues at treatment endpoint and were also similar among all three groups: sedentary 13.7 ± 3, low intensity 15.6 ± 2.5, and moderate intensity 13.5 ± 0.4 nmol/mg in cardiac muscles; sedentary 16.4 ± 2.6, low intensity 17.6 ± 3.5, and moderate intensity 15.6 ± 2.3 nmol/mg in diaphragm muscles; sedentary 25.5 ± 3.2, low intensity 22.0 ± 6.7, and moderate intensity 23.4 ± 7.1 nmol/mg in gastrocnemius muscles (Fig. 4, right). We also quantitated centrally nucleated myofibers in skeletal muscle, a marker of injured/regenerating myofibers, and circulating levels of creatine kinase, a marker of body-wide muscle damage. The percentage of centrally nucleated myofibers did not change among the groups (Supplemental Fig. 3A). Serum creatine kinase did not significantly change during the exercise program (Supplemental Fig. 3B). Taken together, these data indicate that aerobic exercise improved function of dystrophic heart and muscle without significantly increasing fibrosis, centrally nucleated fibers, or serum CK.

### Exercise increased adiponectin and decreased adipocyte cross sectional area in mdx mice

**Figure 5:**
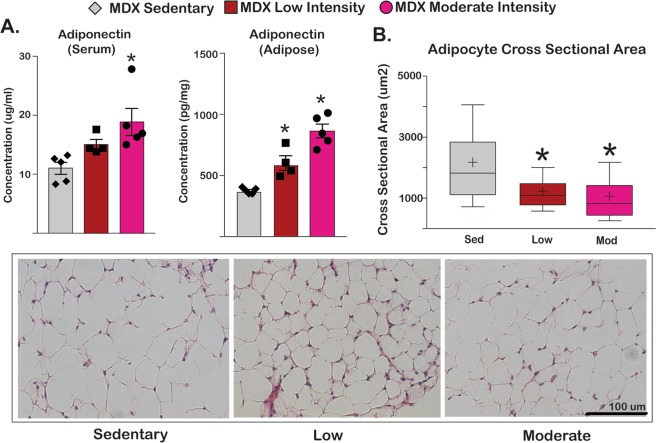
Exercise increased serum adiponectin and decreased adipocyte cross sectional area in *mdx* mice. (**A**) Serum adiponectin levels and adiponectin content in gastrocnemius (GCN) muscle and adipose tissue were increased in the exercise cohorts. (**B**) Adipocyte cross sectional area decreased in exercised *mdx* mice, as quantitated from histological staining of omental fat. Histograms depict single values and mean ± s.e.m.; box plots depict 10–90% distribution with mean (+); *P < 0.05 vs sedentary; 1-way ANOVA test with Tukey’s multiple comparison.

In normal muscle, exercise is generally associated with engagement of adipose tissue. With normal physiology, aerobic exercise increases adiponectin levels both in the circulation and within fat tissue and is a marker for increased fat utilization^17^. Adiponectin levels were assessed in both serum and adipocytes collected from omental fat at the study end. We observed an exercise dose-dependent increase in serum and adipocyte concentrations of adiponectin (Fig. 5A). In serum, sedentary mice had 11.0 ± 1.0 μg/mL, low intensity had 15.1 ± 0.9 μg/mL, and moderate intensity had 18.9 ± 2.3 μg/mL. These same trends were seen in omental adipose tissue (sedentary 376 ± 11 pg/mL, low intensity 602 ± 60 pg/mL, and moderate intensity 864 ± 56 pg/mL). There was a corresponding reduction in the cross sectional area of omental adipocytes in the exercise cohorts compared to the sedentary cohort (Fig. 5B). Adipocytes in the sedentary cohort had an average cross sectional area of 2120 ± 38 μm^2^ whereas the low intensity group had an average cross sectional area of 1211 ± 18 μm^2^ and the moderate intensity cohort had a cross sectional area of 1040 ± 22 μm^2^. Thus, aerobic exercise in dystrophic mice increased adiponectin levels in fat and systemic circulation, and associated with a reduction of adipocyte CSA.

### Moderate intensity treadmill running increased activity in mdx mice

**Figure 6:**
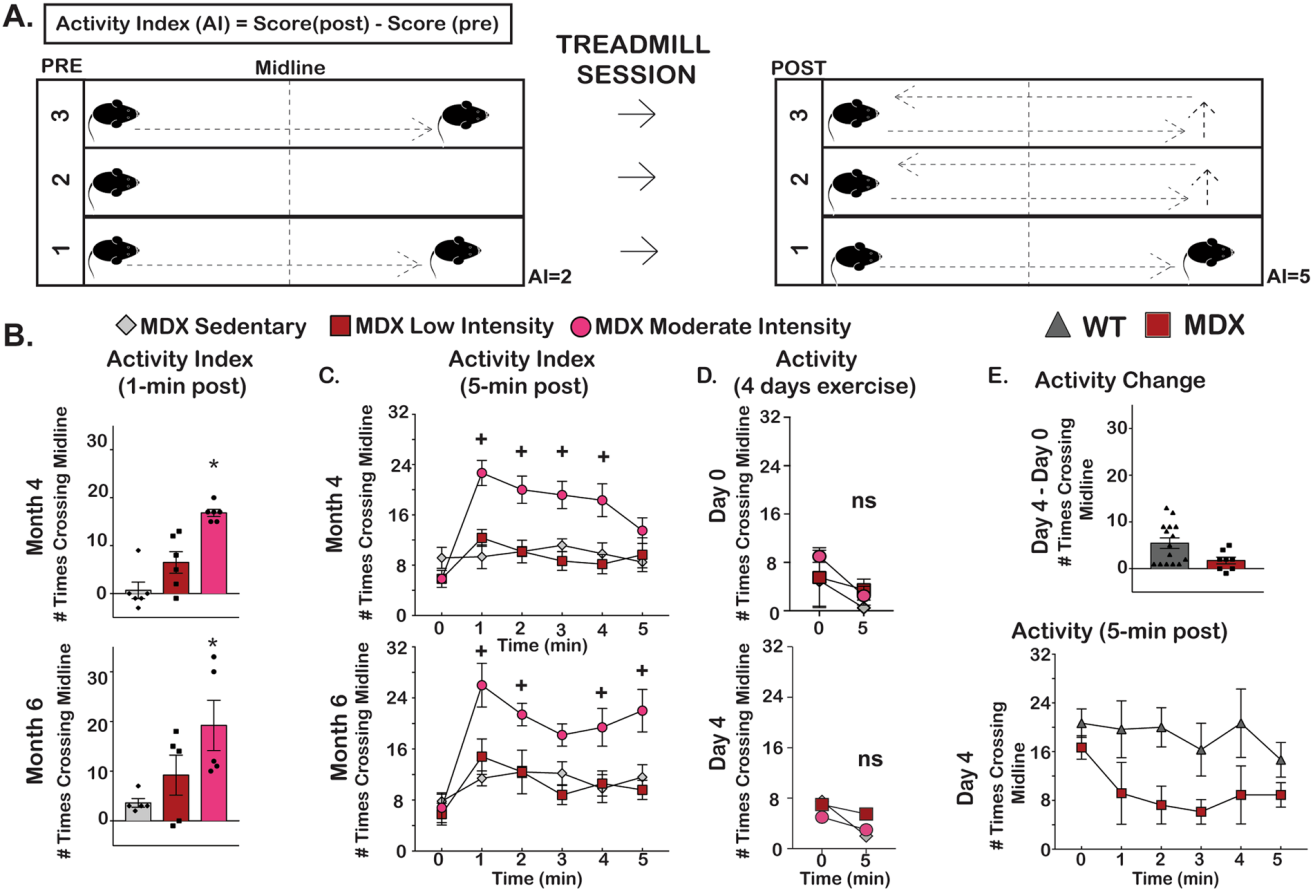
Moderate intensity treadmill running was associated with increased activity in *mdx* mice (**A**) An Activity Index (AI) was used to compare activity before and after treadmill exercise. A score of 1 was given per each time a mouse crossed the treadmill midline, and the score pre-exercise was subtracted from the post-exercise score. (**B,C**) Sustained activity post exercise was only seen in moderate intensity exercise group. (**D**) *mdx* mice from the DBA2J background were subjected to low or moderate intensity exercise over four days, and this exposure to exercise did not alter activity. (**E**) Strain-matched WT and *mdx*-DBA2J mice were exercised over four days using the low intensity protocol. Comparing activity levels after exercise from day 4 to day 0 showed activity was greater in WT mice versus *mdx* mice. Activity was higher in WT than in *mdx* mice when monitored for 5-minutes post-exercise at day 4. Histograms depict single values and mean ± s.e.m.; curves depict mean ± s.e.m.; *P < 0.05 vs sedentary; 1-way ANOVA test with Tukey’s multiple comparison; ns (non-significant), P > 0.05, + , P < 0.05 vs sedentary, 2-way ANOVA test with Tukey’s multiple comparison.

When examining mice in their cages, we noted a subjective increase in activity of those animals exposed to exercise. In order to quantify these changes, we developed an exploratory endpoint, the activity index (AI) (Fig. 6A). We monitored activity defined as number of times crossing track midline (CM) before and after exercise, since post exercise fatigue has been observed in *mdx* mice^18^. During months 4 and 6 of exercise, there was an increase in average AI in the moderate intensity exercise group compared to the sedentary and low intensity exercise group evaluating activity at 1 min pre and post exercise (Fig. 6B). At month 4, the AI for sedentary *mdx* mice was 0.6 ± 1.7 CM, while for low intensity it was 6.5 ± 2.3 CM and for moderate intensity it was 16.8 ± 0.8 CM. Similar trends were seen at month 6 (sedentary 2.0 ± 0.9 CM, low intensity 9.2 ± 4.0 CM, moderate intensity 19.2 ± 5.1 CM.) When considering activity 5 minutes pre and post exercise, only the moderate intensity group had an increase in spontaneous activity after exercising (Fig. 6C). The low intensity exercise group was not different than the sedentary group. To assess the effect of short term exercise on activity, a different cohort of *mdx* DBA/2 J age-matched animals were subjected to low and moderate exercise for 4 days, and post-exercise activity values at 0 and 5 min were compared at day 0 and day 4. No significant change in activity was seen after four days of exercise in *mdx* (Fig. 6D). Thus, the increase in ad libitum activity noted in exercised *mdx* mice appears to be from chronic exposure to exercise.

To compare baseline activity between control and *mdx* mice, 15 wildtype DBA/2 J and eight *mdx* DBA/2 J age-matched animals at eight to ten months of age, were compared over a four day exercise protocol. At day 0 (D0) and day 4 (D4), activity was monitored, and at each time point *mdx* mice had less activity than strain-matched wildtype (Fig. 6E).

## Discussion

The progressive decline of activity in DMD boys is seen most acutely between the ages of 7 and 12, reflected by timed walk testing as well as activity monitors^19–21^. The degree to which to encourage aerobic exercise in DMD, especially in the presence of established disease, remains an open question. Small randomized trials in DMD have suggested benefit with exercise for both lower and upper extremities^22,23^. Furthermore, endurance training in Becker Muscular Dystrophy has been associated with improved fitness^24^. Mortality in DMD often relates to cardiovascular and pulmonary outcomes, indicating the importance of exercise and how it may impact long-term morbidity and mortality^25,26^. Moderate exercise in DMD may be important for cardiovascular and metabolic fitness, provided such exercise does not exacerbate the dystrophic process in skeletal muscle.

We now used the *mdx* model in the DBA2J background to examine physiological consequences of low and moderate intensity exercise. The data presented herein further expands knowledge on effects of exercise on dystrophic muscle pathology through (i) a novel treadmill-based protocol of aerobic exercise with standard speeds; (ii) validating adiponectin measurement as a metabolic biomarker of submaximal exercise effects in dystrophic animals; and (iii) introducing activity index as an exploratory tool to compare pre- and post-exercise activity. These data indicate that submaximal exercise is beneficial in *mdx* mice from the DBA/2J background. We selected this model since it has more fibrosis and less regenerative capacity compared to the more commonly used C57Bl6/10 backgrounds^16,27^. Specifically, the *mdx* model in the DBA2J background is useful for examining cardiac phenotypes and this was seen in this work, as sedentary mice demonstrated a decline in fractional shortening over the time course of this study (~4 months to one year of age). The findings are consistent with a recent study of voluntary wheel and its beneficial effects on cardiomyopathic features of older *mdx* mice on the C57Bl/6 background, as both studies examined the effect of exercise on established disease^28^. We found the decline in LVEF was mitigated in a dose-dependent manner, with the moderate intensity cohort showing significantly delaying the decline of heart function. A limitation of this current study is the absence of background-matched WT mice exposed to same sedentary and exercise regimens, as this comparator would help determine whether the degree of gain achieved by dystrophic mice is similar to that of WT mice. There are no standardized doses of low, moderate, and high intensity exercise for *mdx* mice^29,30^. We considered 12 m/min as a potential high intensity regiment based on the Treat-NMD protocol. We defined low and moderate intensity exercises as 4 m/min and 8 m/min, although it is possible that other regimens of altered speed and duration would similarly show benefit. Human studies are needed to assess appropriate levels of low and moderate exercise intensity and how they may impact skeletal muscle, cardiac, and respiratory function.

Submaximal exercise may favorably alter the hemodynamic and metabolic programming associated with muscle dystrophy. This improvement can manifest as improved muscle function, improved cardiac function, and increased respiratory capacity. As data continues to be collected with regards to muscle disease and exercise, it may have greater implications for additional forms of neuromuscular disease^31^. Defining biomarkers for skeletal and cardiac dystrophic muscle may help better define low and moderate levels of exercise so that these programs can translate into the human setting. It is notable that we observed a dose-dependent increase in the metabolic hormone adiponectin in exercised mdx mice. Where it was previously examined in response to high intensity exercise in *mdx* mice (12 m/min, twice weekly for 12 weeks), adiponectin mRNA levels trended downward^12^, suggesting that adiponectin may be a useful biomarker to reflect a favorable response to exercise in DMD. Adiponectin is reduced in *mdx* mice and DMD patients compared to controls, and upregulation of adiponectin has favorable effects on muscle regeneration^32–34^. Moderate exercise may be an effective means of increasing adiponectin levels.

Based on mutational status, some DMD patients are now receiving antisense-mediated exon skipping drugs^35^ or gene therapy^36^ as part of clinical trials or as FDA approved drugs. The effect of exercise alongside these treatments may potentially have an additive impact in muscle strength and function in addition to delaying respiratory and cardiac decline associated with disease progression.

## Methods and Materials

### Ethics statement

All experiments using living animals in this study were performed in ethical accordance with the American Veterinary Medical Association (AVMA) guideline and under protocols approved by the Institutional Animal Care and Use Committee (IACUC) at Northwestern University Feinberg School of Medicine (protocol number ISO00000761).

### Animal procedures and cohorts

Experiments were performed on male *mdx* mice on the DBA/2 J background from The Jackson Laboratory (Bar Harbor, ME; stock #013141). Three cohorts of mice ages 4 to 5 months old (n = 5 each) were established: moderate intensity (8 m/min), low intensity (4 m/min), and sedentary (0 m/min). All mice were housed in a pathogen free dedicated vivarium and fed with Mouse Breeder Sterilizable Diet (#7904; Harlan Teklad, Indianapolis, IN) and maintained on a 12-hour light/dark cycle. Age-matched wildtype DBA/2 J mice were used as controls (n = 5) and did not undergo exercise protocols. Animals were sacrificed at the end of the 24-week exercise program by carbon dioxide inhalation followed by cervical dislocation. Heart, diaphragm, gastrocnemius, tibialis anterior, and quadriceps were excised immediately. The heart and diaphragm were washed in 10X cardioplegia and then bisected for flash freezing in liquid nitrogen or fixed in formalin for histology.

Baseline exercise studies comparing control and mdx mice used age-matched (8–10 months old) wildtype DBA/2 J and *mdx* DBA/2 J mice for baseline activity index assessment. Fifteen wildtype mice were separated into 3 groups (n = 5): sedentary, low intensity, and moderate intensity. Eight *mdx* mice were separated into three groups: sedentary (n = 3), low intensity (n = 3), and moderate intensity (n = 2). The exercise groups underwent the same protocol as the experimental groups, but for a total of four exercise sessions on consecutive days.

### Exercise protocol

The rodent treadmill Exer 3/6 (Columbus Instruments, USA) was used with opaque dividers separating the six running lanes preventing visualization of other mice during exercise. The electrical shock grid was not used. The treadmill remained horizontal throughout the study. Mice exercised on Tuesday, Wednesday, and Friday of each exercise week between the hours of 8 am–12 pm. The sedentary cohort was placed in the treadmill for 30 minutes at a speed of 0 m/min for the entire session. The low intensity cohort reached a maximum speed of 4 m/min. From start to 2:29, the mice paced at 1 m/min. From 2:30 to 4:59, the mice paced at 2 m/min. From 5:00 to 7:29 the mice paced at 3 m/min. Then from 7:30 to 30:00 the mice ran at max speed of 4 m/min. The moderate intensity cohort reached a maximum speed of 8 m/min. From start to 2:29, the mice paced at 2 m/min. From 2:30 to 4:59, the mice paced at 4 m/min. From 5:00 to 7:29 the mice paced at 6 m/min. Then from 7:30 to 30:00 the mice ran at max speed of 8 m/min. Each cohort (n = 5) was exercised concurrently.

### Activity Index (AI)

Mice were placed on treadmill for 5 minutes prior to beginning exercise to allow for acclimatization. Videos were taken immediately pre and post exercise. Midline was defined with pre-determined markers on the treadmill. Number of times crossing midline (CM) was counted using videos that were captured on an iPad. AI was derived by averaging the total per cohort (n = 5 mice) per exercise session.

### Muscle function

Forelimb grip strength was measured using a meter (Cat #1027SM; Columbus Instruments, Columbus, OH). Mice performed five pulls with 5 seconds rest on a flat surface between pulls on Sunday mornings between 8am-10am every 2 months. At end of study, immediately before sacrifice, *in situ* tetanic force from the right side tibialis anterior muscle was measured using a Whole Mouse Test System (Cat #1300 A; Aurora Scientific, Aurora, ON, Canada) with a 1 N dual-action lever arm force transducer (300C-LR, Aurora Scientific, Aurora, ON, Canada) under anesthesia (0.8 l/min of 1.5% isoflurane in 100% O2). Tetanic isometric contraction was induced with following specifications: initial delay, 0.1 sec; frequency, 200 Hz; pulse width, 0.5 msec; duration, 0.5 sec; using 100 mA stimulation^37^. Length adjusted to fixed baseline of 50 mN resting tension for all muscles/conditions. Fatigue analysis was conducted by repeating tetanic contractions every 10 seconds until complete exhaustion of the muscle (50 cycles).

### Hydroxyproline and creatine kinase (CK) measurements

Hydroxyproline content was measured on 25 mg of gastrocnemius muscle, diaphragm muscle, and heart tissue, as previously described^38^. Briefly, 50 mg pulverized muscle tissue was incubated overnight in 2 ml 6 M HCl at 110 °C. Ten μl of supernatant was combined with 150 μl isopropanol and 75 ml solution A (chloramine T, acetate citrate buffer, 1:4 ratio) and incubated at room temperature for 10 minutes. 1 ml solution B (Ehrlich’s reagent, isopropanol, 3:13 ratio) was added and incubated at 58 °C for 30 minutes. After centrifugation at 5000 rpm for 1 minute, samples were assayed for absorbance at 558 nM. Results were reported as nmol hydroxyproline/mg (tissue) using a standard curve of purified hydroxyproline. Serum CK was analyzed for each mouse using the EnzyChrom Creatine Kinase Assay (Cat # ECPK-100; BioAssay Systems, Hayward, CA). Briefly, every serum sample was diluted 1:10 to a final 10 μl volume, then combined with substrate solution, assay buffer and enzyme buffer (ratio 10:100:1; 100 μl per sample). Experiments were controlled with negative and calibrator controls, optical density was monitored using kinetic measurements at 340 nm, and CK levels were quantitated as U/ml using reads at 20 and 40 minutes. Both hydroxyproline and CK samples were analyzed with the Synergy HTX multi-mode plate reader (BioTek®, Winooski, VT). Both hydroxyproline and CK assays were conducted blinded to treatment groups.

### Fiber typing

Excised *Tibialis anterior* (TA) muscle was placed in an Eppendorf tube and snap frozen in liquid nitrogen and stored at −80 °C until sectioning. Sections were made at 10 μm using a cryostat and tissues were stained. Antibody staining for fiber typing was performed under the following conditions: 4% paraformaldehyde fixation (15 minutes, room temperature); permeabilization with 0.2% Triton (catalog number X-100; Sigma-Aldrich), 1% bovine serum albumin (catalog number A7906; Sigma-Aldrich), and PBS (30 minutes, room temperature); and blocking in 1% bovine serum albumin and 10% fetal bovine serum and PBS (30 minutes, room temperature). Sections were incubated with primary antibodies BA-F8 (1:10), SC-71 (1:30), and BF-F3 (1:10); all from Developmental Studies Hybridoma Bank (Iowa City, IA) overnight at 4 °C. Then, sections were incubated (1:500) with secondary antibodies AlexaFluor350 anti-IgG2b, AlexaFluor488 anti-IgG1, and AlexaFluor594 anti-IgM (catalog numbers A21140, A21121, and 1010111, respectively; Life Technologies). Type 1 fibers stained blue, type 2 A fibers stained green, and type 2B fibers stained red. Immunofluorescence microscopy (IFM) images were captured within 18 hours of completion of secondary staining.

### Whole-body plethysmography (WBP)

Whole-body plethysmography measured respiratory function using a Buxco Finepointe 4-site apparatus (Data Sciences International, New Brighton, MN). Individual mice were placed in a calibrated cylindrical chamber at room temperature without anesthesia between 9 am–12 pm. Each mouse had at least 24 hours of rest from most recent exercise session. Data was recorded for a total of 15 minutes broken into 3 consecutive 5-minute periods. All respiratory studies were conducted blinded to treatment groups.

### Cardiac function

Echocardiography was conducted under anesthesia (0.8 L/min of 1.5% vaporized isoflurane in 100% O2) on a Visual Sonics Vevo 2100 imaging system with an MS550D 22–55 MHz solid-state transducer (FujiFilm, Toronto, ON, Canada). Longitudinal and circumferential strain measurements were calculated using parasternal long-axis and short-axis B-mode recordings of three consecutive cardiac cycles, analyzed by the Vevo Strain software (FujiFilm, Toronto, ON, Canada). Recording and analysis were conducted blinded to treatment group. Electrocardiography (EKG) was conducted without anesthesia on an ECGenie (Mouse Specifics, Inc., Framingham, MA). Mice were acclimated for 5-minutes preceding the 15-minute data collection period. Tracings analyzed in software provided (EzCG Signal Analysis Software). Cardiac mass was assessed at end of study with sacrifice. After excision heart was immediately washed in 10X Cardioplegia buffer to remove any excess blood and weighed.

### Adiponectin

Adiponectin was quantified through mouse-specific Quantikine ELISA kits (#MRP300; R&D, Minneapolis, MN), using kits’ protocols and internal standards to calculate μg/ml (mouse serum) and pg/mg (gastrocnemius muscle and adipose) values. Analysis used either serum or 25 mg of frozen-pulverized tissue (treated according to each kits procedure). Colorimetric reactions were quantified with a Synergy HTX multi-mode plate reader (BioTek®, Winooski, VT) at a wavelength of 570 nm.

### Real-Time Quantitative PCR (RT-PCR)

Total RNA was extracted with Trizol (catalog number 15596018; Life Technologies) from 25 mg tissue. RNA (1.5 μg) was reverse transcribed with the qScript cDNA kit (catalog number 95048; Quanta Biosciences, Beverly, MA), following the kit’s instructions. cDNA was diluted 1:14, and 2 μL was used per 10 μL real-time quantitative PCR (qPCR). Each qPCR contained 100nmol/L primers and 5 μL iTaq SYBR Green Mix (catalog number 1725124; Bio-Rad, Hercules, CA). The CFX96 RealTime System (Bio-Rad) was used to run the qPCR (95 °C, 10 seconds; 57 °C, 30 seconds; 55 cycles) and quantitate fluorescence. Relative fold change among groups was normalized to Rn45s. Primers for PCR:

PGC1a: F1 (5′ CCCACAGAAAACAGGAA 3′); R1 (5′ TGGTTGGCTTTATGAGGA 3′)

Rn45s: F1 (5′ GTAACCCGTTGAACCCCATT 3′); R1 (5′ CCATCCAATCGGTAGTAGCG 3′)

### Histology

Excised tissues (heart, diaphragm, omental fat, and skeletal muscles) were placed in 10% formaldehyde (Cat #245–684; Fisher Scientific, Waltham, MA) for histologic processing. Sections (6 μm) were obtained from the center of paraffin-embedded muscles and stained with hematoxylin and eosin (H&E; cat #12013B, 1070 C; Newcomer Supply, Middleton, WI) or Picrosirius red staining (Cat #P6744–1GA; Sigma-Aldrich; St. Louis, MO). Picrosirius red staining was performed on de-waxed and hydrated paraffin sections after brief staining with Weigert’s hematoxylin (8 minutes), through a one hour incubation in saturated Picrosirius red solution. After two washes in acidified water, sections were de-hydrated, mounted and imaged at ~20% brightfield luminosity with a DIC filter and auto-white settings. Adipocyte CSA quantitation was conducted on ~300 adipocytes per tissue per mouse collected from omental fat. Myofiber CSA quantitation was conducted on approximately 600 myofibers per tissue per mouse. Central nuclei quantitation was performed on ~500 myofibers per treatment group using H&E stained gastrocnemius muscle sections. Picrosirius red staining analysis was performed through automatic threshold analysis using ImageJ^39^. Imaging was performed using a Zeiss Axio Observer A1 microscope, using 10X and 20 × (short-range) objectives. Brightfield pictures were acquired via Gryphax software (version 1.0.6.598; Jenoptik, Jena, Germany). Area quantitation was performed by means of ImageJ^39^.

### Statistical analysis

Statistical analyses were performed using the Prism software v7.0a (Graphpad, La Jolla, CA). When comparing three groups of data for one variable, one-way ANOVA with Tukey multi-comparison was used. When comparing data groups for more than one related variable (typically time and treatment) two-way ANOVA was used and the Tukey multi-comparison additionally used when comparing more than two data groups. For ANOVA and t-test analyses, a P value less than 0.05 was considered significant. Grubbs’ test (alpha = 0.05) was used to identify and remove outliers. Outliers removed: one data point from sedentary group for *Pgc1a* expression analysis in gastrocnemius muscles. Data were presented as single values (dot plots, histograms) when the number of data points was less than 10. Analyses pooling data points over time were presented as marked line plots. Tables, dot plots, histograms and marked line plots depict mean ± SEM or ± SD. Box plots depict the 10–90% distribution of the data pool with mean charted (+).

## Supplementary information

Supplementary Figures

## Data Availability

All primary data will be made available on request.

## References

[1] Guiraud S (2015). The Pathogenesis and Therapy of Muscular Dystrophies. Annu Rev Genomics Hum Genet.

[2] Eagle M (2002). Report on the muscular dystrophy campaign workshop: exercise in neuromuscular diseases Newcastle, January 2002. Neuromuscul Disord.

[3] Wineinger MA, Abresch RT, Walsh SA, Carter GT (1998). Effects of aging and voluntary exercise on the function of dystrophic muscle from mdx mice. American journal of physical medicine & rehabilitation.

[4] Sandri M (1997). Exercise induces myonuclear ubiquitination and apoptosis in dystrophin-deficient muscle of mice. Journal of neuropathology and experimental neurology.

[5] Hulmi JJ (2013). Exercise restores decreased physical activity levels and increases markers of autophagy and oxidative capacity in myostatin/activin-blocked mdx mice. American journal of physiology. Endocrinology and metabolism.

[6] Fraysse B (2004). The alteration of calcium homeostasis in adult dystrophic mdx muscle fibers is worsened by a chronic exercise *in vivo*. Neurobiology of disease.

[7] Dupont-Versteegden EE, McCarter RJ, Katz MS (1994). Voluntary exercise decreases progression of muscular dystrophy in diaphragm of mdx mice. Journal of applied physiology (Bethesda, Md.: 1985).

[8] De Luca A (2003). Enhanced dystrophic progression in mdx mice by exercise and beneficial effects of taurine and insulin-like growth factor-1. The Journal of pharmacology and experimental therapeutics.

[9] Brussee V, Tardif F, Tremblay JP (1997). Muscle fibers of mdx mice are more vulnerable to exercise than those of normal mice. Neuromuscul Disord.

[10] Bizario JC (2009). Imatinib mesylate ameliorates the dystrophic phenotype in exercised mdx mice. J Neuroimmunol.

[11] Anderson CL (2006). The mouse dystrophin muscle promoter/enhancer drives expression of mini-dystrophin in transgenic mdx mice and rescues the dystrophy in these mice. Mol Ther.

[12] Camerino GM (2014). Gene expression in mdx mouse muscle in relation to age and exercise: aberrant mechanical-metabolic coupling and implications for pre-clinical studies in Duchenne muscular dystrophy. Hum Mol Genet.

[13] Fukada S (2010). Genetic background affects properties of satellite cells and mdx phenotypes. Am J Pathol.

[14] Hakim CH (2017). A Five-Repeat Micro-Dystrophin Gene Ameliorated Dystrophic Phenotype in the Severe DBA/2J-mdx Model of Duchenne Muscular Dystrophy. Molecular therapy. Methods & clinical development.

[15] Rodrigues M (2016). Impaired regenerative capacity and lower revertant fibre expansion in dystrophin-deficient mdx muscles on DBA/2 background. Scientific reports.

[16] Coley WD (2016). Effect of genetic background on the dystrophic phenotype in mdx mice. Hum Mol Genet.

[17] Lihn AS, Pedersen SB, Richelsen B (2005). Adiponectin: action, regulation and association to insulin sensitivity. Obes Rev.

[18] Kobayashi YM (2008). Sarcolemma-localized nNOS is required to maintain activity after mild exercise. Nature.

[19] Bello L (2016). DMD genotypes and loss of ambulation in the CINRG Duchenne Natural History Study. Neurology.

[20] Fowler EG (2018). Longitudinal community walking activity in Duchenne muscular dystrophy. Muscle & nerve.

[21] Henricson EK (2013). The cooperative international neuromuscular research group Duchenne natural history study: glucocorticoid treatment preserves clinically meaningful functional milestones and reduces rate of disease progression as measured by manual muscle testing and other commonly used clinical trial outcome measures. Muscle & nerve.

[22] Jansen M, van Alfen N, Geurts AC, de Groot IJ (2013). Assisted bicycle training delays functional deterioration in boys with Duchenne muscular dystrophy: the randomized controlled trial “no use is disuse”. Neurorehabilitation and neural repair.

[23] Alemdaroglu I, Karaduman A, Yilmaz OT, Topaloglu H (2015). Different types of upper extremity exercise training in Duchenne muscular dystrophy: effects on functional performance, strength, endurance, and ambulation. Muscle & nerve.

[24] Sveen ML (2008). Endurance training improves fitness and strength in patients with Becker muscular dystrophy. Brain: a journal of neurology.

[25] Connuck DM (2008). Characteristics and outcomes of cardiomyopathy in children with Duchenne or Becker muscular dystrophy: a comparative study from the Pediatric Cardiomyopathy Registry. Am Heart J.

[26] Benditt JO, Boitano L (2005). Respiratory support of individuals with Duchenne muscular dystrophy: toward a standard of care. Phys Med Rehabil Clin N Am.

[27] Fuller-Thomson E, B Katz R, T Phan V, P M Liddycoat J, Brennenstuhl S (2013). The long arm of parental addictions: the association with adult children’s depression in a population-based study. Psychiatry Res.

[28] Kogelman B (2018). Voluntary exercise improves muscle function and does not exacerbate muscle and heart pathology in aged Duchenne muscular dystrophy mice. J Mol Cell Cardiol.

[29] Kostek MC, Gordon B (2018). Exercise Is an Adjuvant to Contemporary Dystrophy Treatments. Exerc Sport Sci Rev.

[30] Hyzewicz J, Ruegg UT, Takeda S (2015). Comparison of Experimental Protocols of Physical Exercise for mdx Mice and Duchenne Muscular Dystrophy Patients. J Neuromuscul Dis.

[31] Ng SY, Manta A, Ljubicic V (2018). Exercise Biology of Neuromuscular Disorders. Appl Physiol Nutr Metab.

[32] Hathout Y (2015). Large-scale serum protein biomarker discovery in Duchenne muscular dystrophy. Proceedings of the National Academy of Sciences of the United States of America.

[33] Hathout Y (2014). Discovery of serum protein biomarkers in the mdx mouse model and cross-species comparison to Duchenne muscular dystrophy patients. Hum Mol Genet.

[34] Abou-Samra M (2015). Involvement of adiponectin in the pathogenesis of dystrophinopathy. Skeletal muscle.

[35] Charleston JS (2018). Eteplirsen treatment for Duchenne muscular dystrophy: Exon skipping and dystrophin production. Neurology.

[36] Duan D (2018). Micro-Dystrophin Gene Therapy Goes Systemic in Duchenne Muscular Dystrophy Patients. Hum Gene Ther.

[37] Quattrocelli M (2015). Mesodermal iPSC-derived progenitor cells functionally regenerate cardiac and skeletal muscle. J Clin Invest.

[38] Heydemann A (2009). Latent TGF-beta-binding protein 4 modifies muscular dystrophy in mice. J Clin Invest.

[39] Schneider CA, Rasband WS, Eliceiri KW (2012). NIH Image to ImageJ: 25 years of image analysis. Nat Methods.

