# Supplementary material for: Moderate exercise improves function and increases adiponectin in the mdx mouse model of muscular dystrophy
Source: Sci Rep. 2019 Apr 8;9:5770. doi: 10.1038/s41598-019-42203-z (PMC6453911; doi:10.1038/s41598-019-42203-z)
Supplement: Supplementary file 1 — Supplementary Figures [file 41598_2019_42203_MOESM1_ESM.pdf]

SUPPLEMENTARY INFORMATION FOR

Moderate exercise improves function and increases adiponectin in the *mdx* mouse model of muscular dystrophy

Aaron S. Zelikovich, Mattia Quattrocelli, Isabella M. Salamone, Nancy L. Kuntz, Elizabeth M. McNally

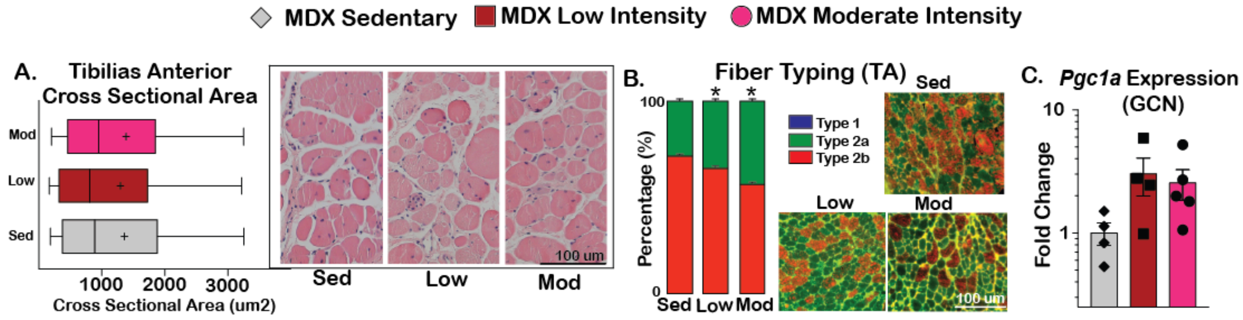

Supplemental Figure 1. Exercise improved skeletal muscle function in *mdx* mice. (A) TA cross sectional area was calculated from histological analysis and did not differ between exercise groups. **(B)** Exercise groups showed an incremental increase in the ratio of type 2a fibers (green) suggesting an adaptive response to oxidative capacity in the TA muscle. **(C)** Exercise groups showed a modest increase in *Pgc1a* gene expression in the gastrocnemius muscle, as quantitated by qPCR analysis of muscle tissue.

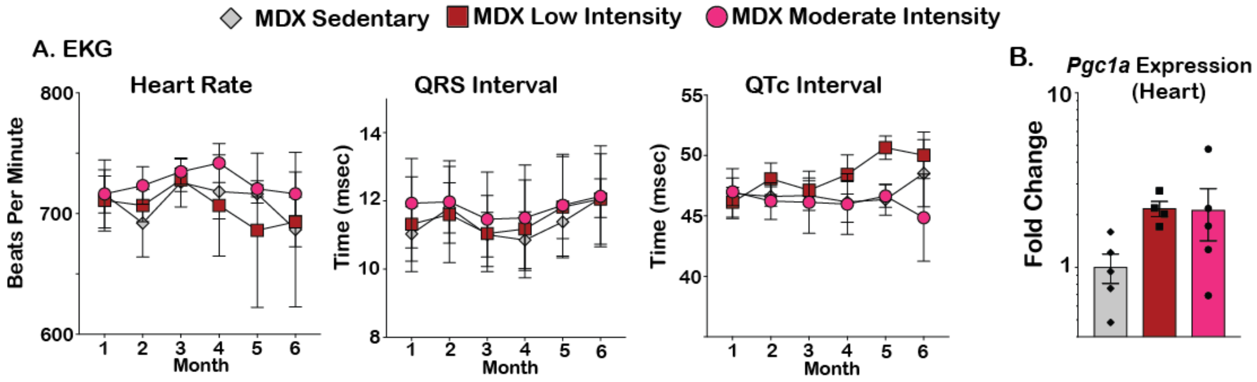

Supplemental Figure 2. Exercise did not change EKG parameters and associated with a modest increase in *Pgc1a* expression in the heart. (A) Heart rate, QRS, and QTc intervals were unchanged by exercise and were similar among the groups. **(B)** *Pgc1a* mRNA expression showed a modest increase in both exercise groups, as compared to the sedentary group.

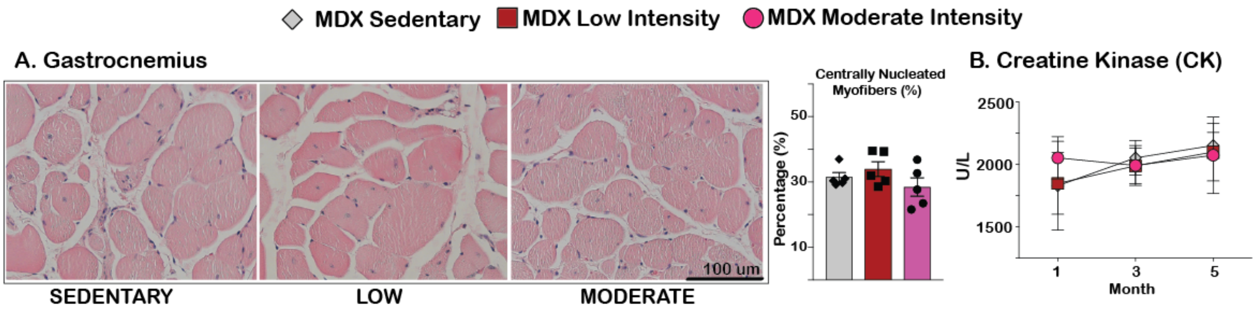

Supplemental Figure 3. Exercise did not increase fraction of centrally nucleated myofibers or serum creatine kinase in *mdx* mice. (A) Exercise did not increase the percentage of centrally nucleated myofibers in gastrocnemius muscle. **(B)** Serum CK levels remained similar in all groups throughout study suggesting that muscle membrane damage was not further increased by exercise.
